# Feasibility of engineered *Bacillus subtilis* for use as a microbiome‐based topical drug delivery platform

**DOI:** 10.1002/btm2.10645

**Published:** 2024-01-02

**Authors:** Veronica A. Montgomery, Amy J. Wood‐Yang, Mark P. Styczynski, Mark R. Prausnitz

**Affiliations:** ^1^ Wallace H. Coulter Department of Biomedical Engineering at Emory University and Georgia Tech Georgia Institute of Technology Atlanta Georgia USA; ^2^ School of Chemical and Biomolecular Engineering Georgia Institute of Technology Atlanta Georgia USA

**Keywords:** *Bacillus subtilis*, engineered live biotherapeutics, skin microbiome, topical drug delivery

## Abstract

Non‐adherence to medication is a major challenge in healthcare that results in worsened treatment outcomes for patients. Reducing the frequency of required administrations could improve adherence but is challenging for topical drug delivery due to the generally short residence time of topical formulations on the skin. In this study, we sought to determine the feasibility of developing a microbiome‐based, long‐acting, topical delivery platform using *Bacillus subtilis* for drug production and delivery on the skin, which was assessed using green fluorescent protein as a model heterologous protein for delivery. We developed a computational model of bacteria population dynamics on the skin and used its qualitative predictions to guide experimental design choices. Using an *ex vivo* pig skin model and a human skin tissue culture model, we saw persistence of delivered bacteria for multiple days and observed little evidence of cytotoxicity to human keratinocyte cells *in vitro*. Finally, using an in vivo mouse model, we found that the delivered bacteria persisted on the skin for at least 1 day during every‐other‐day application and did not appear to present safety concerns. Taken together, our results support the feasibility of using engineered *B. subtilis* for topical drug delivery.

**Translational Impact Statement**

This manuscript presents the first study to address the feasibility and safety of using engineered *B. subtilis* for topical drug delivery to the skin. *B. subtilis* is a highly genetically tractable organism with an established record of safe use in humans, so use of this organism could make the development of bacteria‐based topical delivery vehicles more straightforward and accessible.

## 1 INTRODUCTION

Improving patient adherence to existing medications is a major challenge in healthcare that, due to its wide‐reaching nature, could yield greater health benefits than improvements to any specific medication.^1^ In fact, nonadherence accounts for an estimated 10% of drug‐related hospital admissions, and proper adherence to medication is <50% in countries with advanced economies; it is expected to be much lower in other parts of the world.^2^

Improved drug delivery platforms have the potential to make medication adherence easier for patients by minimizing side effects, allowing more comfortable administration routes, and reducing the number of required administrations.^3^ In the case of dermatology, topical drug delivery platforms are attractive because the therapeutic can be delivered directly to the site of action (i.e., the skin) in a non‐invasive way, typically by patients themselves. This approach avoids the use of needles and other barriers to obtaining treatment, which can help minimize hospital visits and unwanted side effects.

Despite the advantages of topical drug delivery, it is difficult to deliver drugs topically for an extended time. Gels, ointments, and lotions often require daily or more frequent application because of their limited residence time on the skin due to removal by clothing, sweating, rubbing and other effects.^4^ The need for frequent administration of topical delivery formulations can negatively impact patient adherence, especially in the case of chronic diseases such as psoriasis and atopic dermatitis.^5^

An exciting area emerging in the field of drug delivery is the use of bacteria as drug delivery vehicles.^6^, ^7^ The symbiotic relationship between bacteria and the humans they colonize combined with existing technology to engineer bacteria to produce heterologous molecules creates an opportunity to use engineered bacteria as a form of *in situ* drug production and delivery. A growing body of work has reported the design of engineered bacteria to produce therapeutic molecules in the gut, vaginal tract, skin, and mouth.^6^, ^7^, ^8^

In the context of dermatology, there are preclinical and clinical‐stage companies currently working to develop engineered bacteria as topical drug delivery agents. Notable examples include Azitra, which has multiple preclinical products using engineered *Staphylococcus epidermidis* to treat skin disorders^9^; Ilya Pharma, which has developed a clinical‐stage strain of *Limosilactobacillus reuteri* expressing the chemokine CXCL12 to promote wound healing^8^, ^10^; and Xycrobe Therapeutics, which is developing a preclinical strain of *Cutibacterium acnes* engineered to deliver IL‐10 for acne vulgaris.^8^

In this study, we investigate the potential use of engineered *Bacillus subtilis* as a topical drug delivery platform. *B. subtilis* may have advantages over other candidate bacteria as a platform for drug delivery to the skin because of its safety profile and genetic tractability. It is found in the skin microflora and is metabolically active on the skin.^11^, ^12^ It is nonpathogenic and has natural antimicrobial properties against pathogenic staphylococci and fungi.^13^, ^14^, ^15^ *B. subtilis* has generally regarded as safe status from the FDA, and multiple *B. subtilis* probiotic products as well as a genetically modified strain of *B. subtilis* are currently commercially available.^7^, ^16^, ^17^

An important characteristic of *B. subtilis* is that it is commonly used in biotechnology for the production of proteins, vitamins, and antibiotics because of its efficient protein secretion system, ease of cultivation, and high genetic tractability.^18^ As a result, many tools and libraries have been developed for its genetic manipulation, making the design and implementation of engineered *B. subtilis*‐based biotherapeutics more straightforward than the similar use of other, non‐model organisms.^18^, ^19^

Motivated by these characteristics of *B. subtilis*, we sought to assess the feasibility of using *B. subtilis* as a topical drug delivery platform in terms of the duration of drug production on skin and safety. To facilitate characterization, we used an engineered strain of *B. subtilis* producing green fluorescent protein (GFP) as an easily detectable, model heterologous protein. Through a combination of experimental studies supported by computational modeling, we identified that microbiome‐based drug delivery via *B. subtilis* is feasible, with certain constraints.

## 2 RESULTS

### 2.1 Survival and production of heterologous protein by *B. subtilis* on skin

#### 2.1.1 Model simulation

**FIGURE 1:**
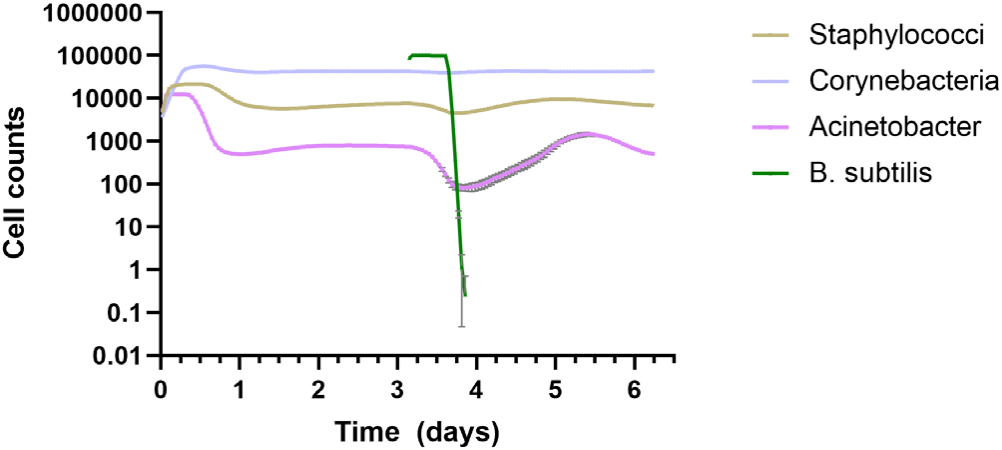
Simulation of skin bacteria population dynamics using an agent‐based model. Population representing *Bacillus subtilis* was added on day 3.125. Lines represent mean values and error bars represent standard deviation of three simulations. In most cases, the error bars are smaller than the line thickness.

In our overall approach to assess the viability of *B. subtilis*‐based skin drug delivery, we relied on a hybrid computational and experimental approach where initial model predictions helped to guide experiments. We first developed an agent‐based model for bacterial population dynamics on skin based on the Gutlogo model of population dynamics in the gut.^20^ This model predicted that the populations representing *Corynebacteria*, *Staphylococci*, and *Acinetobacter* would reach a steady state comprising about 84% *Corynebacteria*, 15% *Staphylococci*, and 1% *Acinetobacter*. When *B. subtilis* was introduced at timestep 4500 (3.125 days), it survived for about half a day before quickly dying off, after which the other species largely returned to their previous steady‐state levels (Figure 1).

#### 2.1.2 Experimental findings

Before assessing survival time of *B. subtilis* on skin experimentally, we first wanted to account for the possibility that *in situ* production of a therapeutic for delivery could have a substantial growth burden on the cells, providing a negative selection pressure. We sought to identify the impact on cell growth of expressing a heterologous reporter protein from a plasmid using GFP as a simple model protein, which could represent either a protein directly serving as a therapeutic or an enzyme used to synthesize a small‐molecule therapeutic.

Using reporter plasmids transformed into *B. subtilis*, we tested six different ribosome binding site (RBS) candidates expected to yield a variety of expression levels to determine if there were expression‐dependent effects on cell fitness. Using doubling time, fluorescence in culture, and fluorescence in co‐culture with the parent (non‐fluorescent) strain to monitor cell fitness, we identified that GFP expression had small (albeit significant) effects on cell fitness (Figure S1). Different drug products could result in larger growth defects than what we observed with GFP, and this will likely vary significantly between different drugs. To assess the possible effects that a larger growth defect might have on survival time, we used the computational model to explore the survival of *B. subtilis* with a 10%, 25%, 50%, and 100% growth defect and found no significant difference to survival time (Figure S2).

To make later measurements of *B. subtilis* survival and protein expression as sensitive as possible, we used RBS5 in strain *BS‐GFP* for experiments moving forward, as it yielded almost an order of magnitude more GFP expression than any other RBS with minimal additional impact on cell growth. We validated that for the timescale of our proof‐of‐concept experiments, the stability of pGFP‐RBS5 in *B. subtilis* in the absence of antibiotics in liquid culture was such that the plasmid was maintained for at least 24 h (Figure S3).

**FIGURE 2:**
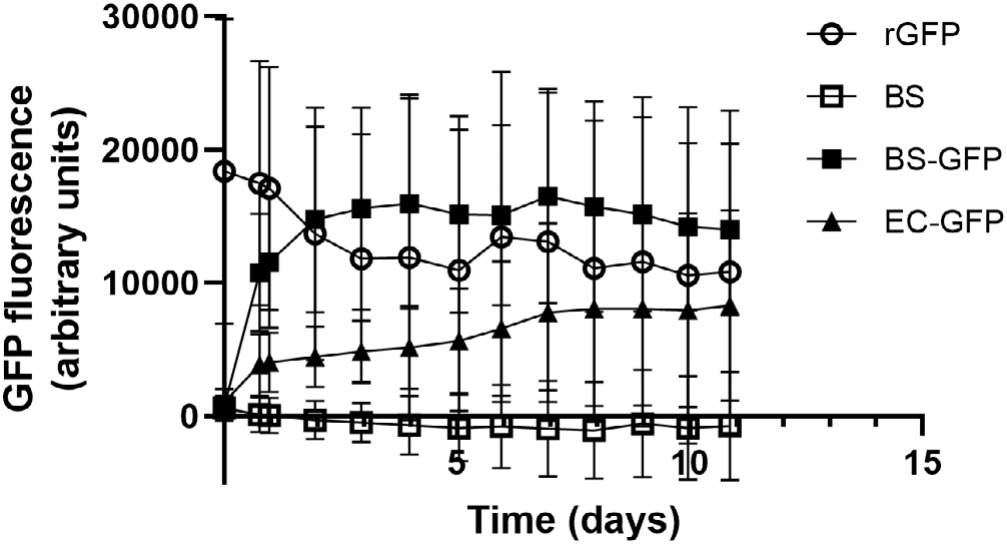
Fluorescence of GFP expressed by *Bacillus subtilis* over time in an *ex vivo* pig skin model. rGFP, recombinant GFP added to skin surface at time 0; BS, *B. subtilis* strain 168; *BS‐GFP*, *B. subtilis* strain 168 harboring pGFP‐RBS5 plasmid; *EC‐GFP*, *Escherichia coli* strain DH5α harboring pGFP‐RBS5 plasmid. Data show mean +/− standard deviation of eight replicates.

We used the resulting strain as the basis for experiments in an *ex vivo* pig skin model. On pig skin that had microorganisms cleaned from the surface (i.e., cleansed, but not sterile), the *BS‐GFP* strain apparently survived on the skin and produced GFP for about 4 days, after which fluorescence reached a plateau (Figure 2). This duration of survival was different from that seen in the model simulation, probably because there was only limited competition with other bacteria on the cleansed pig skin. Comparing *B. subtilis* with *Escherichia coli*, a gram‐negative species with limited survival on human skin^21^ but similar or faster growth rates in liquid culture, we found that *BS‐GFP* produced more GFP than *EC‐GFP* in the pig skin model (two‐way ANOVA, *p* < 0.0001) even though *EC‐GFP* had higher fluorescence expression than *BS‐GFP* in liquid culture (Table S1). Taken together, these results support the potential viability of *B. subtilis* as a skin drug delivery platform.

### 2.2 Effect of added antibiotics

A way to extend the lifetime of added *B. subtilis* in a skin microbiome may be to add a selective pressure that provides an advantage for *B. subtilis* compared to the native microbiome. We investigated one way of forcing a selective advantage: adding an antibiotic to the environment to which *B. subtilis* is resistant but the rest of the microbiome is sensitive.

#### 2.2.1 Model simulation

**FIGURE 3:**
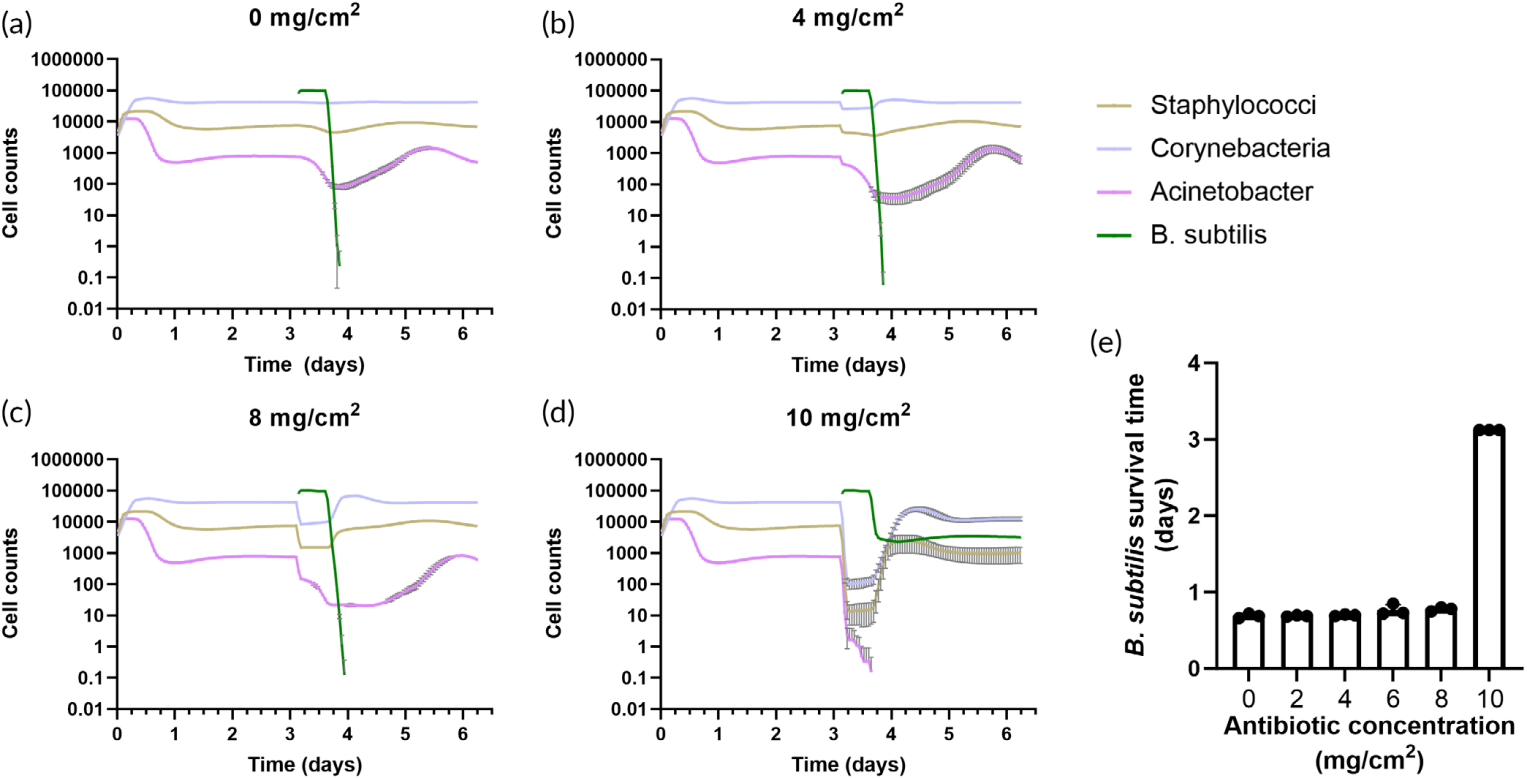
Simulation of skin bacteria population dynamics when adding antibiotics concurrently with *Bacillus subtilis*. (a) 0 mg/cm^2^; (b) 4 mg/cm^2^; (c) 8 mg/cm^2^; (d) 10 mg/cm^2^ of antibiotic. (e) Survival time of *B. subtilis* with different concentrations of antibiotic. At 10 mg/cm^2^, *B. subtilis* appeared to survive long‐term, but is shown as 3 days, because the simulation ended at that point. Lines and bars show mean +/− standard deviation of three replicate simulations.

We first used the computational model to simulate the potential impact of adding such an antibiotic. According to the model, antibiotic concentrations <1000 times the defined MIC for the native microbiome species had almost no effect on the survival of *B. subtilis*, with the other populations recovering shortly after the *B. subtilis* population died. At the highest concentration tested *in silico* (1000 times the defined MIC for the native microbiome species), the *B. subtilis* population appeared to achieve long‐term survival, at the expense of the *Acinetobacter* population dying completely (Figure 3).

#### 2.2.2 Experimental findings

**FIGURE 4:**
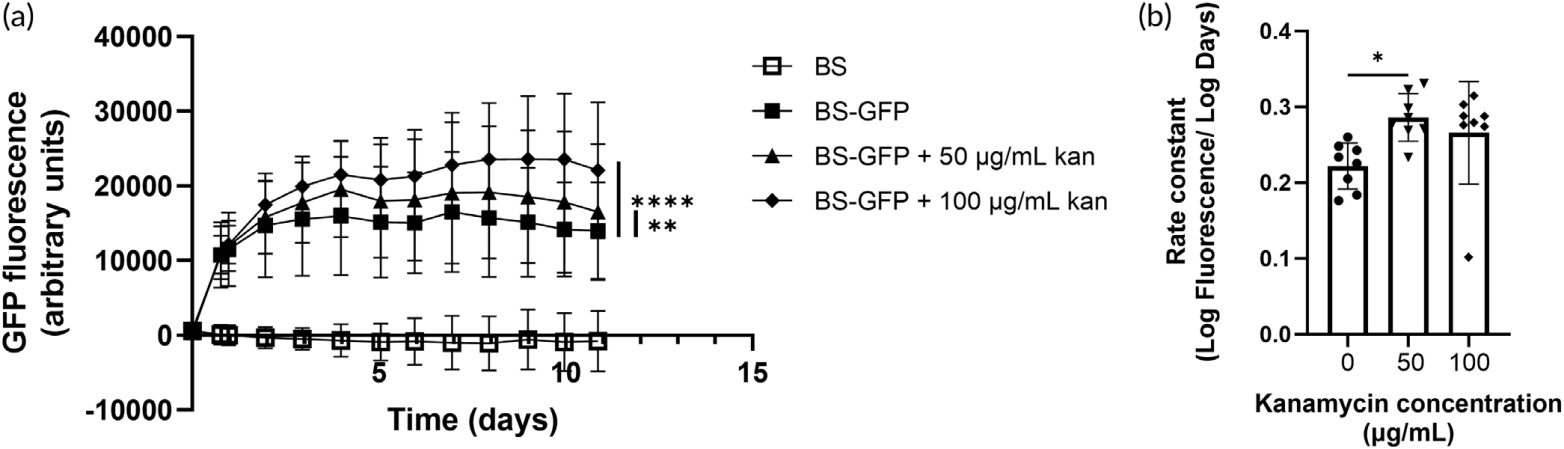
Green fluorescent protein (GFP) production in *ex vivo* pig skin model with antibiotic addition. (a) Fluorescence measurements over time. BS: *Bacillus subtilis* strain 168; *BS‐GFP*: *B. subtilis* strain 168 harboring pGFP‐RBS5 plasmid; *BS‐GFP* + 50 μg/mL kan: BS‐GFP with 50 μg/mL kanamycin added immediately before bacteria; *BS‐GFP* + 100 μg/mL kan: *BS‐GFP* with 100 μg/mL kanamycin added immediately before bacteria. ** *p* < 0.01, **** *p* < 0.0001, two‐way ANOVA compared to *BS‐GFP*. (b) Rate constant of fluorescence curves shown in part (a). * *p* < 0.05, one‐way ANOVA compared to *BS‐GFP*. Data show mean +/− standard deviation of eight replicates.

We next tested the effects of antibiotics in the *ex vivo* pig skin model using the antibiotic kanamycin, to which the *BS‐GFP* strain has resistance due to the selection marker on the plasmid. We found that there was a dose‐dependent effect of antibiotic on total GFP production, as well as an increase in the length of time over which GFP was produced from 4 to 5 days when antibiotic was added (Figure 4). Consistent with the model simulation, the dose‐dependent antibiotic effect may be due to decreased competition with residual microorganisms on the cleansed skin. Coupled with nutrient limitations, this may have led to increased growth of the *B. subtilis* cells and thus higher overall fluorescence. It is possible that the effect of the antibiotic was also due to decreased competition with *B. subtilis* cells that spontaneously lost plasmid and would otherwise have had a growth advantage by virtue of not producing heterologous protein. Again, coupling this with nutrient limitations would lead to more plasmid‐bearing *B. subtilis* and thus higher GFP expression levels.

We characterized the GFP production curves (Figure 4a) with a rate constant (Figure 4b). There was a significant increase in the rate constant of the fluorescence curve for the lower antibiotic concentration and a trend toward an increased rate constant for the higher antibiotic concentration that was not significant (Figure 4b). Taken together, these results suggest that using an antibiotic adjuvant for bacterial‐based drug delivery to skin could have positive impacts on total dosage and persistence of delivery vehicle viability.

### 2.3 Effect of added carbon source

Another way to extend the lifetime of added *B. subtilis* in a skin microbiome may be to supplement a nutrient source that *B. subtilis* would have a distinct advantage in using compared to the native microbiome. It has previously been shown that creation of an exclusive metabolic niche in the gut can lead to long term colonization of a probiotic in mice.^22^ We hypothesized that creating a similar niche on the skin may yield similar results.

#### 2.3.1 Model simulation

**FIGURE 5:**
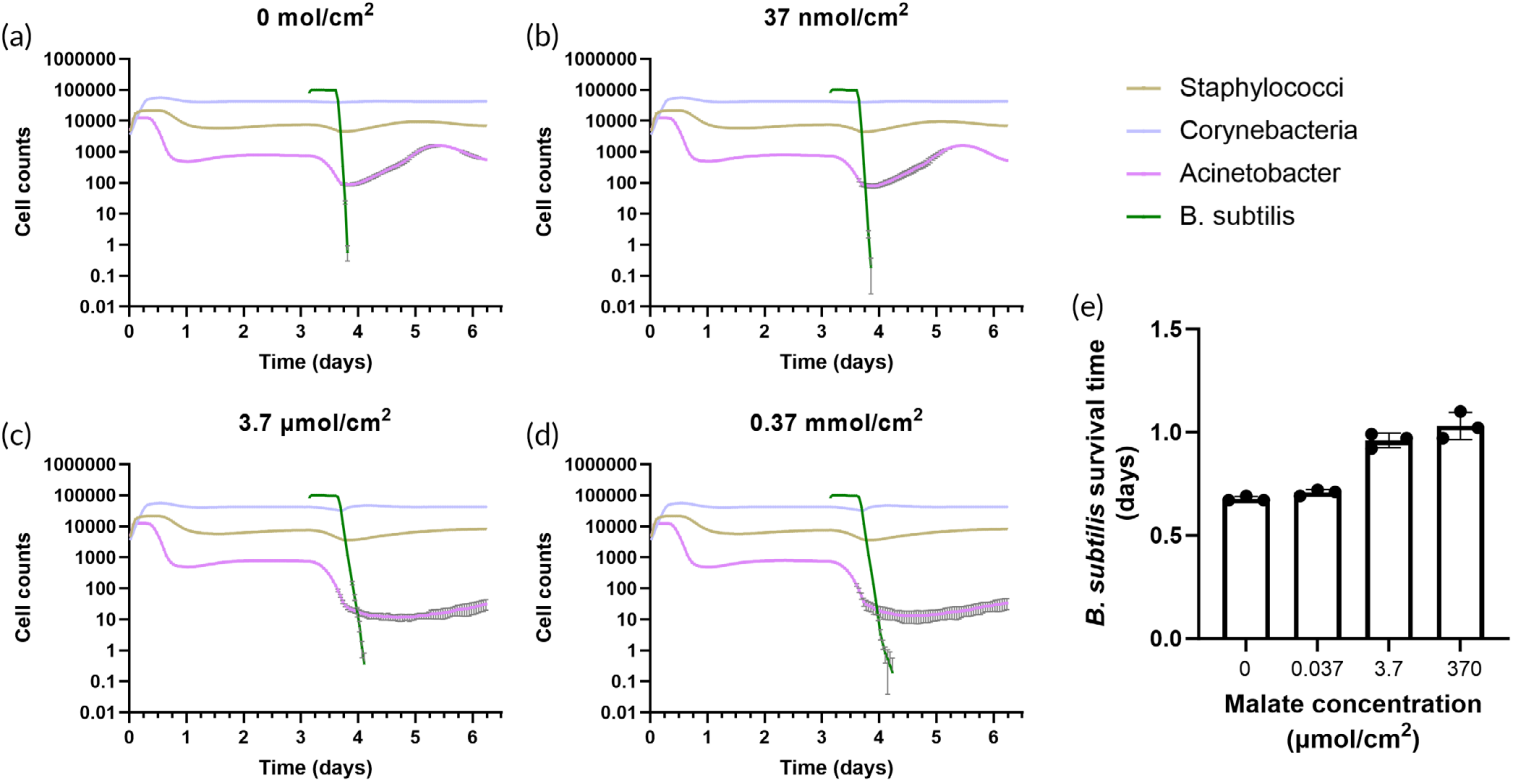
Simulation of skin bacteria population dynamics when adding supplemental carbon source concurrently with *Bacillus subtilis*. (a) 0 mol/cm^2^ malate added; (b) 37 nmol/cm^2^ malate; (c) 3.7 μmol/cm^2^ malate; (d) 0.37 mmol/cm^2^ malate. Lines represent mean values and error bars show standard deviation across three simulations. In most cases, the error bars are smaller than the line thickness. (e) survival time of *B. subtilis* with different concentrations of carbon source. Data show mean +/− standard deviation of three replicate simulations.

We thus used the computational model to evaluate the potential effect of adding a carbon source to specifically benefit *B. subtilis*. We chose to use malate as the additional carbon source; while malate can be consumed by other species, it is a preferred carbon source for *B. subtilis* (in addition to glucose), so we would expect malate addition to most benefit *B. subtilis*. Since the model does not differentiate between preferred and non‐preferred carbon sources, we modeled this supplementation as the creation of an exclusive niche where no other species could use malate as a carbon source. Addition of increasing concentrations of malate led to an increase in predicted *B. subtilis* survival time from about half a day to about 1 day (Figure 5). We also tested the effect of adding additional, non‐specific, nutrients such as LB at the same time as *B. subtilis* and observed a slight increase in survival time (Figures S4 and S5).

#### 2.3.2 Experimental findings

**FIGURE 6:**
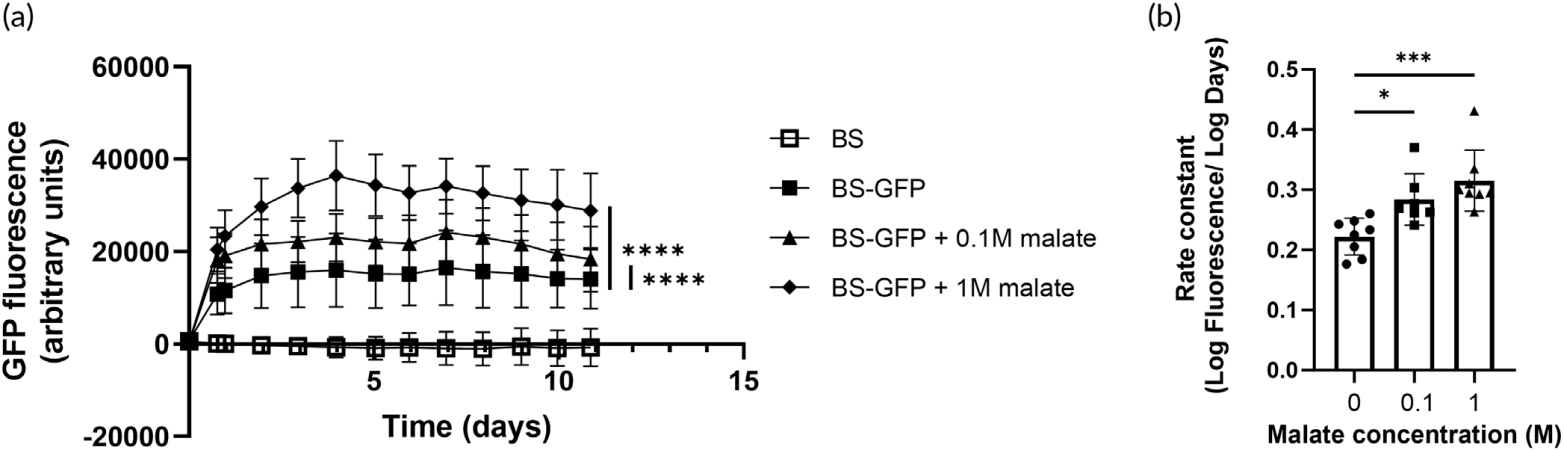
Green fluorescent protein (GFP) production in *ex vivo* pig skin model with malate addition. (a) Fluorescence measurements over time. BS: *Bacillus subtilis* strain 168; *BS‐GFP*: *B. subtilis* strain 168 harboring pGFP‐RBS5 plasmid; *BS‐GFP* + 0.1 M malate: *BS‐GFP* with 0.1 M malate added immediately before bacteria; *BS‐GFP* + 1 M malate: *BS‐GFP* with 1 M malate added immediately before bacteria. **** *p* < 0.0001, two‐way ANOVA compared to *BS‐GFP*. (b) Rate constant of fluorescence curves shown in part (a). * *p* < 0.05, *** *p* < 0.001, one‐way ANOVA compared to 0 M. Data show mean +/− standard deviation of eight replicates.

Malate was added to the *ex vivo* pig skin model at two doses (0.1 and 1 M, which correspond to ~3.6 and 36 μmol/cm^2^, respectively). We observed a dose‐dependent effect of the added malate on total GFP production. We also observed an increase in the length of time over which GFP was produced to about 5 days for the 1 M malate group, as well as significant increases in the rate constants for both malate concentrations (Figure 6). Thus, malate could serve as a useful added nutrient source for *B. subtilis* on skin, enabling increased protein production and a longer duration of protein production.

### 2.4 Survival of *B. subtilis* on human skin culture *in vitro*

#### 2.4.1 Experimental findings

**FIGURE 7:**
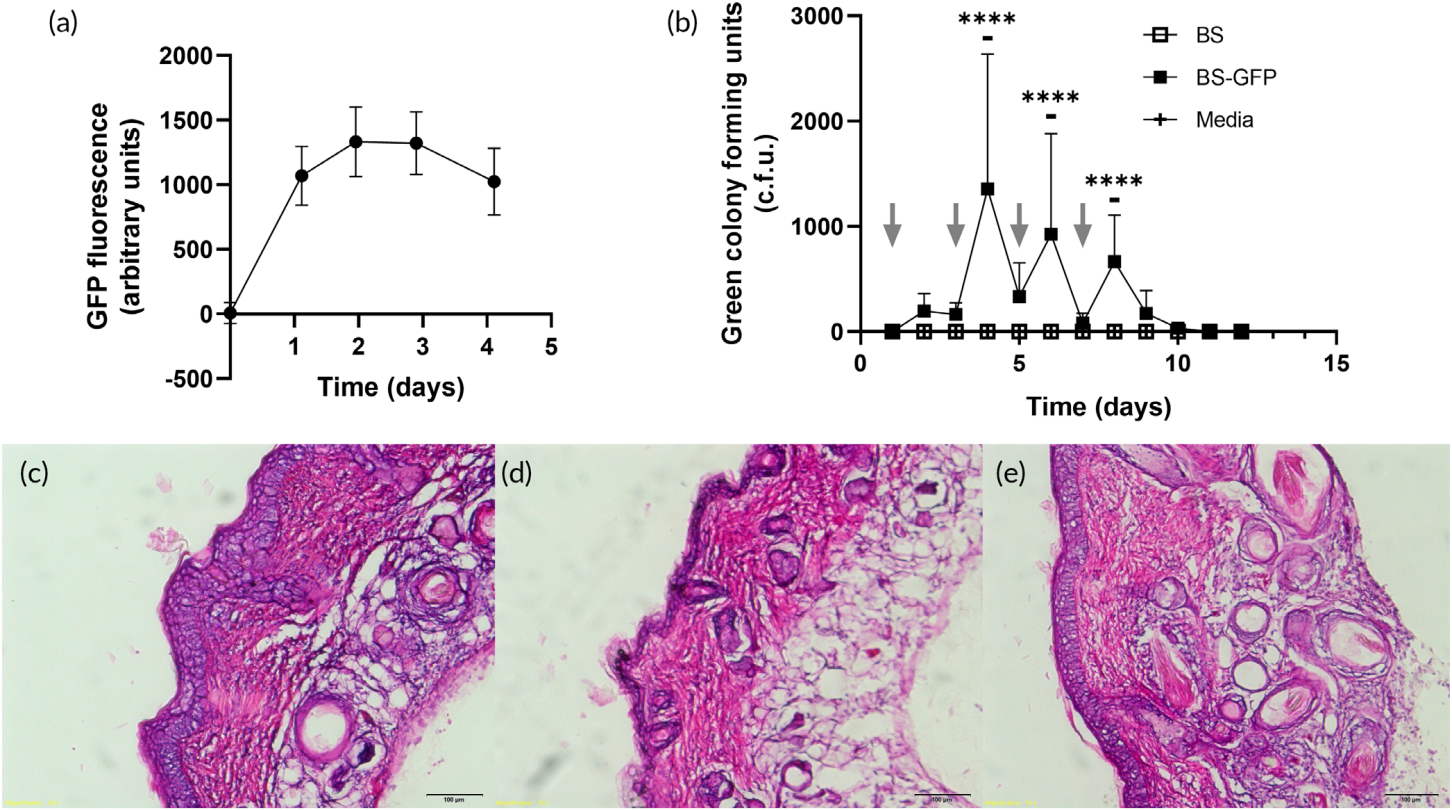
Safety and survival of *BS‐GFP* on human skin *in vitro* and mouse skin in vivo. (a) GFP production in *in vitro* human skin tissue culture model by *BS‐GFP*. Data show mean +/− standard deviation of six replicates. (b) Persistence of *BS‐GFP* on mouse skin in vivo. Bacterial or medium solutions were added every other day (indicated by arrows), and mice were swabbed daily. BS: *B. subtilis* strain 168; *BS‐GFP*: *B. subtilis* strain 168 harboring pGFP‐RBS5 plasmid; Media: LB medium. **** *p* < 0.0001, two‐way ANOVA comparing *BS‐GFP* to media. Data show mean +/− standard deviation of three mice per treatment and three technical replicates of swab plating for each mouse. (c–e) Representative images of mouse skin stained with hematoxylin and eosin. (c) treated with *B. subtilis* strain 168; (d) treated with *B. subtilis* strain *BS‐GFP*; (e) treated with LB medium. Mouse skin in vivo treated with *B. subtilis* or media as shown in (b) was biopsied on day 13, sectioned, stained, and imaged by brightfield microscopy.

Guided by evidence of *B. subtilis* survival on pig skin, we next used a full thickness human skin tissue culture model (Epiderm FT, from MatTek) to assess the ability of *B. subtilis* to survive and produce GFP on human skin that had been precolonized with microorganisms from a swab of human forearm skin (Figure S6). Using fluorescence measurements to track GFP production, we found that *BS‐GFP* survived and produced GFP for 2.1 ± 0.7 days. (Figure 7a), which is generally consistent with the findings from our pig skin model.

### 2.5 Safety of *B. subtilis* on human skin cells *in vitro*

#### 2.5.1 Experimental findings

For *B. subtilis* to be a viable vehicle for therapeutic delivery to the skin, it must not be toxic to the human cells that it will encounter. The potential toxicity of *B. subtilis* was thus tested against HaCaT human keratinocyte cells by exposing the keratinocytes to *B. subtilis* culture supernatants as part of the keratinocyte growth medium. We tested two strains of *B. subtilis*, the common laboratory strain 168 and the antifungal lipopeptide‐producing strain 15841, using exposure to LB media alone as a negative control. We expressed the LD_50_ in terms of the percentage of the supplemented keratinocyte medium composed of LB or supernatant.

The LD_50_ of LB and supernatants from 168 and 15841 cells were 64.0% (95% CI: 49.8–90.50), 33.5% (95% CI: 31.6–35.5) and 28.6% (95% CI: 26.2–31.4), respectively, after 3 days of exposure (Figure S7). The LD_50_ values for both strains 168 and 15841 were significantly different from that of LB media (*p* < 0.0001), which means that the supernatants of both strains had greater toxicity than the media alone but were of the same order of magnitude.

### 2.6 Safety and survival of *B. subtilis* on mouse skin in vivo

#### 2.6.1 Experimental findings

Given that the *in vitro*, *ex vivo*, and computational model results suggest the viability and safety of *B. subtilis* on skin, we used a mouse model in vivo for further validation. *BS‐GFP*, the parent strain *B. subtilis* 168, and LB medium were inoculated onto the backs of hairless mice every other day, with daily swabbing to track whether *BS‐GFP* was still present on mouse skin by counting green colonies after plating on selective medium. We found that *BS‐GFP* was consistently detected on the day following application and sometimes on the following day (Figure 7b).

After the last application of bacteria, *BS‐GFP* was detectable by swab for 3 days and then returned to negligible levels. Mice were then sacrificed, and biopsy samples of skin exposed to bacteria or media were taken for histological analysis. No evidence of tissue damage or infiltrating immune cells were observed for the mice receiving *B. subtilis* treatment (*BS* and *BS‐GFP*) compared to mice treated with just medium (Figure 7c–e).

## 3 DISCUSSION

For topical drug delivery, medications are commonly applied to the skin via gels, ointments, lotions, creams, and sprays, which are advantageous for their ease of application over the affected area of skin.^23^ However, topical formulations are also easily removed from the skin after a short amount of time and often require daily or more‐frequent application to be effective.^4^ Continuous production of a drug or other molecule of interest on skin throughout the day could support enhanced adherence and better outcomes with these forms of treatment. Skin‐resident bacteria like *B. subtilis* could be engineered to continuously produce drugs on the skin for lasting therapy.

Across our various models, we found that *B. subtilis* survived on the skin while producing GFP for at least 1 day and appeared to be safe. Although *B. subtilis* is found on healthy skin, we wanted to confirm that doses of interest for therapeutic applications would not be harmful to the skin, even if at levels much higher than typically found on human skin.^12^ While we found that the supernatants of the two *B. subtilis* strains tested were more toxic to human keratinocyte cells than just LB medium alone, the LD_50_ values were similar, leading us to conclude that the strains would likely be safe for use on skin, but more work is needed to fully answer this question. Future studies incorporating real drugs would need to test the cytotoxicity of the engineered strain in addition to the parent strain to account for any drug‐related toxicity. Furthermore, no signs of negative skin reaction were observed on mouse skin in vivo after 1 week of every‐other‐day application of *B. subtilis*. Altogether, our data suggest that *B. subtilis* may be well tolerated by skin, at least at the levels used in this study.

A safety component which was not experimentally investigated in this study was the effect of *B. subtilis* treatment on the existing microbiome. The microbial community present on the skin is associated with skin health, so it would be important to also ensure that any changes to the existing community are not harmful.^24^ It is worth noting that the computational model predicted that, without antibiotic addition, after the delivered species dies off the existing community would return to its previous levels. This suggests that perturbations to the skin microbiome due to the use of *B. subtilis* as a delivery vehicle may just be transient. In agreement with this observation, other work analyzing the shift in mouse ear microbiota composition during and after *B. subtilis* administration found that there were shifts in the microbiota composition during administration of the bacteria, but the microbiota composition was similar to that of the untreated control group within a week after stopping administration of bacteria.^25^

Starting with equal numbers of the representative skin microbiome species in this model, the relative steady state percentages predicted by the computational model qualitatively matched relative percentages of their genera identified from swabs of human forearm skin.^26^ The computational model also predicted that a species with doubling time and carbon source utilization characteristics representative of *B. subtilis* could survive within an established community representing the skin microbiome for a little over half a day. It predicted that adding a carbon source to specifically benefit the added species could lead to a modest increase in survival time and that application of an antibiotic to which the added species has resistance could lead to long‐term survival, although with long‐term consequences to the existing community. These predictions generally aligned with experimental results using an *ex vivo* pig skin model, where we found that the engineered strain of *B. subtilis* survived on skin while producing GFP for multiple days, and the addition of either kanamycin, to which the engineered strain had resistance, or malate, a preferred carbon source for *B. subtilis*, led to significantly increased GFP production. While addition of antibiotics increased *B. subtilis* survival in both models, the FDA has recommended against the use of antibiotic selection markers in clinical stage products.^27^ We used antibiotics in this study to develop a proof‐of‐concept understanding of *B. subtilis* survival on skin in the presence of selective survival pressures, but in a future human study or commercial product using antibiotics as a selective pressure would not be suitable. Alternative selective pressures, such as added nutrients that favor *B. subtilis* growth, would be a preferred approach.

We used a full thickness human skin tissue culture model to evaluate the survival of *B. subtilis* on human skin in the presence of microorganisms obtained from human forearm skin and found that *BS‐GFP* survived while producing GFP for about 2 days. While this model provided further evidence that *B. subtilis* can survive on human skin, we applied a relatively low density of human skin microorganisms to the tissue surface, so further studies would be required to further determine the ability of *B. subtilis* to survive in the context of the skin microbiome. Moreover, our inclusion of LB in the delivery vehicle may have provided nutrients for *B. subtilis* survival not typically present on the skin and thus affecting cell growth; however, this approach was useful to maintain consistency across experimental models, and additives could be used in a final formulation if the additional nutrients prove critical to enabling this approach.

Finally, when testing survival of *B. subtilis* on mouse skin in vivo, we found that *BS‐GFP* was repeatedly detectable by swab 1 day after application, which was generally consistent with the computational model predictions. After applications ceased, *BS‐GFP* was still detectable by swab for 2 days but returned to baseline within 3 days of the last application. A study in which *B. subtilis* was applied to mouse ears for seven consecutive days found that *B. subtilis* levels returned to baseline levels within 4 days after the last application, which is in general agreement with our results.^25^ Taken together, these findings led us to conclude that *B. subtilis* could be suitable as a drug delivery platform for a daily or every‐other‐day application but may not be suitable for longer‐term delivery.

Wild‐type *B. subtilis* may be a more transient member of the skin microbiome compared to other skin‐resident bacteria,^28^ such that longer‐resident species, such as *S. epidermidis*, may be more suited to multiday delivery scenarios.^29^ For example, the company, Azitra, has multiple clinical and preclinical stage products incorporating engineered *S. epidermidis* to treat various skin disorders.^9^ However, variable colonization efficacy has remained a challenge in the field of topical probiotics utilizing resident skin commensal species including *S. epidermidis*, possibly due to competition with the existing microbiota or variability in the skin sites within and between individuals.^30^ Furthermore, due to biocontainment concerns, a common strategy to prevent transfer of the engineered strain to undesired areas is to render the strain an auxotroph requiring regular application of an additional substance, such as d‐alanine, to maintain viability.^31^ We detected *B. subtilis* using fluorescent measurements and growth on selective agar plates, but it is possible that residual cells could persist on the skin that have lost the ability to produce recombinant protein, which would result in an underestimation of *B. subtilis* viability on the skin. This possibility warrants further investigation, and biocontainment would need to be considered for *B. subtilis* as well, likely employing a similar strategy to the engineered auxotrophy strategy currently used in industry. With these considerations in mind, the genetic tractability of *B. subtilis* makes it an attractive option for situations in which daily or every‐other‐day application is appropriate.

A limitation of this study is that GFP production represents a base case scenario in which recombinant expression of a model protein had little effect on cellular fitness, whereas this may not be the case for *in situ* biosynthesized therapeutics. Small‐molecule drugs that require complex pathways for production could require expression of multiple heterologous genes and/or the biosynthetic reactions could affect cellular metabolism,^32^ which would likely affect cell growth and ability to survive on skin. Even protein therapeutics may still have deleterious effects on cellular fitness, especially when produced at high levels. On the other hand, our model still used plasmid‐borne expression, whereas genomic insertion, as would likely be done in a final translational implementation, would decrease fitness defects by removing the burden of plasmid maintenance.^32^, ^33^, ^34^ Nonetheless, for implementation of *in situ* biosynthesis of any therapeutic, the specific toxicity and defects associated with making that specific product, as well as the impacts of that product on the host's natural microbiome, would need to be studied and accounted for in the final design of the organism or delivery vehicle.

Also, logistical considerations of human routines such as hand and body washing and transfer of bacteria from skin to other surfaces could limit the potential applications of using engineered bacteria as a topical drug delivery platform.

## 4 MATERIALS AND METHODS

### 4.1 Computational model

We adapted the code for Gutlogo, an agent‐based model made in NetLogo to simulate population dynamics of the gut microbiota, to represent the skin environment.^20^ The basic rules of Gutlogo were preserved in our skin model, but with the flow component removed, species characteristics adjusted to reflect a set of common skin microbiome constituents, and metabolite concentrations adjusted to reflect expected conditions on the skin. Malate was considered a carbon source for only *B. subtilis*. Additional model details are available in the Data S1. Code for our model, Skinlogo, is publicly available at https://github.com/gtStyLab/skinlogo.git.

### 4.2 Bacterial culture

Strains and plasmids used in this study are listed in Table 1. *B. subtilis* 168 and *E. coli* DH5α were cultured in lysogeny broth (LB), consisting of 0.5% w/v yeast extract (Life Technologies, Carlsbad, CA), 1% w/v NaCl, and 1% w/v tryptone (Life Technologies), at 37°C with shaking at 200 rpm. Growth was measured by absorbance at 600 nm. Fluorescence and absorbance were measured with a Biotek Synergy H4 Hybrid Microplate Reader (Agilent, Santa Clara, CA). For fitness comparisons, each strain containing a different ribosome binding site (RBS) was inoculated into a co‐culture with equal volume of the parent strain *B. subtilis* 168 in LB media without antibiotics, and fluorescence was compared with that of the engineered strain in monoculture. Fluorescence was normalized by absorbance at 600 nm, and the normalized fluorescence values after 24 h in culture were averaged for three replicates.

**TABLE 1:** DNA plasmids and bacterial strains.

Name	Description	Reference/source
DNA plasmids		
pRB374	*Escherichia coli*–*Bacillus subtilis* shuttle plasmid; amp^r^, kan^r^	ATCC^37^
pGFP‐RBSX	pRB374; vegII: *GFP*; containing ribosome binding sites 0–5 (*X* = 0–5)	This study
Bacterial strains		
*DH5α*	*E. coli* high efficiency competent cells	NEB
*EC‐GFP*	DH5α carrying pGFP‐RBS5	This study
168	*B. subtilis* parent strain	ATCC
15841	Antifungal lipopeptide‐producing strain of *B. subtilis*	ATCC^38^
*BS‐GFP*	168 carrying pGFP‐RBS5	This study

Abbreviations: ATCC, American Type Culture Collection (Manassas, VA); NEB, New England Biolabs (Ipswich, MA).

### 4.3 Plasmid assembly

DNA primers used in this study are listed in Table S5. All plasmids were assembled in *E. coli* DH5α and then transformed into *B. subtilis* 168. *E. coli* cells were transformed by heat shock following the NEB high efficiency transformation protocol (New England Biolabs, Ipswich, MA). *B. subtilis* cells were transformed by electroporation using an ECM 600 electroporator (BTX, Holliston, MA) set to 2.1 kV, 129 Ω, and 50 μF with 1 mm gap cuvettes. Superfolder GFP was synthesized by Eurofins (Louisville, KY). GFP was inserted into pRB374 by Gibson assembly, resulting in plasmid pGFP‐RBS0.^35^ For RBS analysis, each RBS was incorporated into the forward primer for amplification of GFP, which was inserted into pRB374 by Gibson assembly, resulting in the plasmids pGFP‐RBSX (*X* = 1–5). To assess plasmid stability, *BS‐GFP* was grown in LB media with or without selecting antibiotic (10 μg/mL kanamycin). Fluorescence over time was compared between the two growth conditions and was normalized by absorbance at 600 nm. The average value of three replicates was reported.

The plasmid pGFP‐RBS5 was used to express GFP in *E. coli* and *B. subtilis* for *ex vivo* and in vivo experiments (*EC‐GFP* and *BS‐GFP*, respectively). During strain and plasmid construction, *E. coli* and *B. subtilis* were grown in LB media with 100 μg/mL carbenicillin and 10 μg/mL kanamycin, respectively, at 37°C.

### 4.4 Mammalian cell culture

HaCaT cells, an immortalized keratinocyte cell line originally obtained from a male patient (AddexBio, San Diego, CA), were cultured in Dulbecco's Modified Eagle Medium (4.5 g/L glucose, 1 mM sodium pyruvate, 4 mM l‐glutamine, ThermoFisher, Waltham, MA) containing 10% v/v fetal bovine serum (FBS, ThermoFisher) and 1% v/v penicillin–streptomycin (ThermoFisher). Cell cultures were grown in a humidified incubator set to 37°C and 5% CO_2_.

### 4.5 LD_50_ cytotoxicity assay

HaCaT cells were seeded in 96‐well plates at a density of 1 × 10^3^ cells per well and incubated for 3 days at 37°C and 5% CO_2_ in a humidified incubator. After 3 days, the medium in the wells was replaced with keratinocyte media containing either LB medium or sterile‐filtered supernatant from overnight *B. subtilis* 168 or 15841 cultures at concentrations ranging from 0.00019% to 50% v/v, with eight replicates per condition. Bacterial supernatants were adjusted to pH 7 with 1 M NaOH before being added to mammalian cells. Plates were incubated another 3 days, after which the medium was replaced with sterile phosphate‐buffered saline (PBS), 10 μL alamarBlue (ThermoFisher) was added to the wells, and plates were incubated for 1–4 h at 37°C.

Wells were read using a Biotek Synergy H4 Hybrid Microplate Reader according to the assay protocol. For the positive toxicity control, 2% Tween 20 was added to the cell culture media to kill the cells. For the negative toxicity control, no supernatant was added to the cell culture media. Additional details about the alamarBlue assay are available in Data S1.

### 4.6 Preparation of bacteria for skin model experiments

Overnight cultures were diluted in LB and grown with shaking at 200 rpm and 37°C with appropriate antibiotics (100 μg/mL carbenicillin for *E. coli* and 10 μg/mL kanamycin for *B. subtilis*) until they reached an absorbance at 600 nm wavelength of ~0.6. Cells were then diluted 1:10 in fresh LB with appropriate antibiotics to be inoculated onto skin models.

### 4.7 Pig skin model

Six millimeter biopsy punches were taken from *ex vivo* pig ear inner pinna skin (Pel‐freez, Rogers, AR). The skin pieces were rinsed in 0.1% v/v peracetic acid (Pfaltz & Bauer, Waterbury, CT) diluted in sterile PBS, adjusted to pH 7.0–7.4 with 1 M NaOH, for 3 h to clean microorganisms from the skin surface.^36^ The skin was then rinsed in two washes of sterile PBS for a total of 30 min to remove residual peracetic acid. Skin pieces were loaded into an HTS Transwell 96‐well Permeable Support plate with a black receiver plate (8 μm pore size, Corning), with sterile PBS in the bottom wells refilled daily to maintain moisture in the skin.

Bacteria were inoculated onto the surface of the skin pieces as 10 μL of *BS‐GFP* or *EC‐GFP* in LB, with eight replicates per experimental group. For the malate and kanamycin experiments, the added bacteria were supplemented with L‐malic acid (10 μL of either 0.1 M or 1 M in sterile PBS, Eastman, Kingsport, TN), which was neutralized to pH 7.0 with 5 M NaOH before application to skin, or kanamycin (10 μL of 50 μg/mL or 100 μg/mL in sterile PBS). *B. subtilis* 168 in LB and sterile PBS were used as negative controls, and 100 μg/mL rGFP (VWR, Radnor, PA) in sterile PBS was used as a positive control. Plates were stored at 32°C, with lids raised slightly up from the plate to prevent condensation on the skin pieces.

Fluorescence was measured at 475 nm excitation and 510 nm emission wavelengths using a fluorescence area scan with matrix size of 5 × 5, set to gain 70, with a Biotek Synergy H4 Hybrid Microplate Reader. The average fluorescence value of the sterile PBS group was subtracted as background at each time point. The derivatives of the resulting fluorescence curves were estimated using a central difference approximation. GFP production was considered to have stopped when the derivative became negative.

Rate constants were determined by plotting GFP fluorescence values over time for individual samples on a log–log scale and taking the average slope of the trendlines. We interpret a greater rate constant to indicate a greater GFP production rate.

### 4.8 Human skin tissue culture model

Full thickness human skin tissue cultures grown from keratinocytes and fibroblasts obtained from Caucasian neonatal male foreskin were obtained from MatTek (Epiderm FT model, Ashland, MA). Upon arrival, skin tissues were equilibrated at 37°C and 5% CO_2_ with antibiotic‐ and antifungal‐free media provided by MatTek. Skin tissue culture media was changed daily, per the manufacturer's protocol.

Because the skin tissue culture model is sterile, we added microorganisms from a human skin swab to the surface of the tissue culture to create a skin microbial community. After equilibration of the tissue culture model, 20 μL of human skin swab solution was applied to the surface of the skin tissue. For human skin swabs, a sterile HydraFlock swab (Puritan, Guilford, ME) was soaked in sterile PBS with 0.1% v/v Tween 20 and gently rubbed on the forearm skin of a Caucasian female volunteer for about 30 s. This use of the human volunteer was approved by the Georgia Institute of Technology Institutional Review Board with informed consent. The flocked tip of the swab was then broken off into the same tube of sterile PBS with 0.1% v/v Tween 20 and stored for use. The same volume of swab solution (20 μL) was plated on LB agar to monitor microbial load (Figure S6).

After inoculation with human skin swab solution, skin tissue cultures were incubated at 37°C and 5% CO_2_ for 24 h, at which time 20 μL of either LB media or *BS‐GFP* solution was applied to the tissue surface (six samples per group). For fluorescence measurements, cassettes holding the skin tissue culture samples were transferred to a 6‐well plate with black tape lining the walls of the individual wells. Fluorescence was measured with 475 nm excitation and 510 nm emission wavelengths using a fluorescence area scan with matrix size of 5 × 5, set to gain 70, with a Biotek Synergy H4 Hybrid Microplate Reader. The average fluorescence value of the LB media group was subtracted at each time point. The derivative of the resulting fluorescence curve for *BS‐GFP* was estimated using a central difference approximation. GFP production was considered to have stopped when the derivative became negative.

### 4.9 Mouse model

Experiments with mice were approved by the Georgia Tech Institutional Animal Care and Use Committee under protocol number A100427. Six‐ to eight‐week‐old male SKH1 hairless mice from Charles River (Wilmington, MA) were used for in vivo mouse experiments. A Covance Infusion Harness (Instech, Plymouth Meeting, PA) was used to minimize grooming at the application site once bacteria were applied to the skin. The harnesses provided a covered dome over the application site on the back of the mouse and remained on the mouse for the duration of the experiment, enabling application of bacteria and swabbing at the same location on the mouse at each time point. While the harnesses were fitted to the mice, they were not fixed in place, so movement of the harnesses could have potentially influenced bacterial density at the application site. Mouse toenails were clipped at the start of the study and once per week thereafter to prevent tampering with the harnesses. Mice were housed individually to prevent contamination and tampering with harnesses.

Two days before the start of the experiment, the back of each mouse was cleaned with an isopropyl alcohol wipe and fitted with a harness. Mice were randomized into groups by assigning them numbers based on the location of their cage and then grouping them into treatment groups (three treatment groups composed of three mice each) according to their numbers. Test solutions of either *BS‐GFP*, *B. subtilis* 168, or LB alone (20 μL) were applied to the protected area of skin under the harness every 2 days, with daily skin swabs under the protected area of the harness to determine survival of bacteria. For swabbing, a sterile HydraFlock swab was soaked in sterile PBS with 0.1% v/v Tween 20 and gently rubbed on the skin for about 30 s. The flocked tip of the swab was then broken off into the same tube of sterile PBS with 0.1% v/v Tween 20 and stored for plating.

Swab samples were plated on LB agar with 10 μg/mL kanamycin as well as LB agar with no antibiotic to monitor the bacterial load of swabs. Presence of *BS‐GFP* in swabs was determined by counting green colony forming units (c.f.u.) on kanamycin plates. Daily swabbing continued until c.f.u. counts were negligible. Mice were then sacrificed, and a 5 mm biopsy skin sample was taken from the area covered by the harness for histological analysis.

### 4.10 Histology

Mouse skin was stored in 10% neutral buffered formalin for at least 24 h after harvesting. Samples were then transferred to optimal cutting temperature compound (ThermoFisher) and frozen on dry ice. Skin sections measuring 10 μm thick were taken using a Cryostar NX70 cryostat (ThermoFisher) with blade and block temperature set to −20°C. Sections were hematoxylin and eosin stained with an Autostainer XL (Leica, Wetzlar, Germany).

### 4.11 Statistical analysis

Results are presented as mean +/− standard deviation. Two‐way ANOVA followed by Dunnett's multiple comparisons test was used to compare differences of fluorescence in the pig skin model. One‐way ANOVA followed by Dunnett's multiple comparisons test was used to compare rate constants, LD_50_ values, fluorescence in liquid culture, and doubling times. Doubling times were calculated by fitting the mid‐exponential phase of the growth curves to an exponential growth equation. Statistical analyses and linear regressions were performed using GraphPad Prism version 9.4.1 for Windows (San Diego, California, www.graphpad.com). The *p*‐values <0.05 were considered significant.

## 5 CONCLUSION

In this study, we assessed the feasibility of using *B. subtilis* as a topical drug delivery platform. Using GFP as a model heterologous protein, we found that engineered *B. subtilis* could survive on multiple skin models for at least 1 day and appeared to be safe for skin. Due to its wide commercial use and relative ease of genetic modification, we believe that *B. subtilis* could be an attractive candidate to make the development of bacteria‐based topical delivery platforms more straightforward and accessible.

## AUTHOR CONTRIBUTIONS

**Veronica A. Montgomery:** Conceptualization (equal); investigation (lead); methodology (lead); writing – original draft (lead); writing – review and editing (equal). **Amy J. Wood‐Yang:** Investigation (supporting). **Mark P. Styczynski:** Conceptualization (equal); resources (equal); supervision (equal); writing – review and editing (equal). **Mark R. Prausnitz:** Conceptualization (equal); funding acquisition (equal); resources (equal); supervision (equal); writing – review and editing (equal).

## FUNDING INFORMATION

This work was supported in part by an American Fellowship from the American Association of University Women, a National Science Foundation Graduate Research Fellowship (DGE‐1650044), and a Tau Beta Pi Graduate Fellowship to VAM and a National Institutes of Health (R01‐EB034301) to MPS. AJWY was supported by the Georgia Tech Summer Undergraduate Research in Engineering program.

## CONFLICT OF INTEREST STATEMENT

The authors have no conflicts of interest to declare.

### PEER REVIEW

The peer review history for this article is available at https://www.webofscience.com/api/gateway/wos/peer-review/10.1002/btm2.10645.

## Supporting information


**Data S1.** Supplementary Information.

## Data Availability

All data are available in the article or supporting information. Code for our model, Skinlogo, is publicly available at https://github.com/gtStyLab/skinlogo.git.
