# Supplementary material for: Feasibility of engineered Bacillus subtilis for use as a microbiome‐based topical drug delivery platform
Source: Bioeng Transl Med. 2024 Jan 2;9(4):e10645. doi: 10.1002/btm2.10645 (PMC11256169; doi:10.1002/btm2.10645)
Supplement: Supplementary file 1 — Data S1. Supplementary Information. [file BTM2-9-e10645-s001.docx]

**Supporting Information**

**Table S1.** Normalized fluorescence in liquid culture. Fluorescence and absorbance were measured after liquid culture before diluting samples 10-fold and applying to skin models.

| BS-GFP | EC-GFP |
| --- | --- |
| 45362 +/- 1779 | 77345 +/- 487 |

**Computational Model**

We used an agent-based model to predict the survival of bacteria on the skin because of the flexibility of this modeling approach in situations where detailed mathematical knowledge of the governing equations is incomplete [1, 2]. In agent-based modeling, individual agents are defined with a set of rules that determine their behavior, and the model simulates interactions between different agents [1].

The basic rules of Gutlogo were preserved in our model. Each agent represents a bacterium that can ‘eat’ a set of defined carbon sources. Each agent has an energy level that starts at 100 and decreases at each time step down to 0 within a simulated day if it does not eat. Agents with an energy level of 0 die, and agents with an energy level above 50 divide based on their age and a defined doubling time at which their energy level is halved and a new agent of the same species with half the energy level is created. Agents with an energy level below 80 are considered ‘hungry’; on each time step (representing one minute), the model runs through a list of the hungry bacteria and the available nutrients. If a nutrient that is present is a carbon source for a hungry bacterium, the bacterium will consume that nutrient, the number of available units of that nutrient will decrease by 1, and the energy level of the bacterium increases by the energy number assigned to that nutrient. The model incorporates stochasticity through random placement of bacteria in the modeling space, random assignment of each bacterium’s age when the simulation is initiated, and a random selection process through which hungry bacteria are matched with available nutrients.

Beyond these basic principles, other aspects of the model were adjusted to better reflect the skin microbiome. The original Gutlogo model includes a flow component to simulate movement through the gut, which we removed for modeling the skin. We modified the species represented in the model to be reflective of the skin microbiome, and we changed the types and amounts of carbon sources to reflect those found on human forearm skin in previously reported literature [3, 4]. We also included the effects of antibiotics in the model. The endogenous populations were defined as having a minimum inhibitory concentration (MIC) of 10 µg/cm^2^ antibiotic. If there was greater than 10 µg/cm^2^ antibiotic around a bacteria agent, it died, and the amount of antibiotic in the region decreased by 10 µg/cm^2^. The added, engineered *B. subtilis* population was modeled as having no sensitivity to the antibiotic.

The agents in our model comprised three genera of bacteria that are commonly found in the skin microbiome at different abundances, consisting of endogenous *Corynebacteria,* S*taphylococci,* and *Acinetobacter*, as well as our introduced *B. subtilis* [5]. The model was initiated with equal starting numbers of the endogenous populations, and *B. subtilis* was introduced after they appeared to reach steady state (3.125 days). Cell doubling times were based on reported doubling times of representative species in nutrient-rich media (**Table S2**). Carbon sources for each type of bacteria were approximated according to reported carbon source utilization for representative species (**Table S2**). For the purpose of hypothesis generation in the model, malate was considered a carbon source for only the *B. subtilis* agents because malate is a preferred carbon source for *B. subtilis* that has been shown to selectively recruit *B. subtilis* compared to other soil bacteria when secreted from plant roots [6, 7].

**Table S2**. Bacterial agents in model

| **Bacteria Agent Name** | **Doubling Time** | **Carbon Sources** | **References** |
| --- | --- | --- | --- |
| *Acinetobacter* | 40 | Alanine, histidine, proline, phenylalanine, tyrosine | [8, 9] |
| *Corynebacteria* | 90 | Glucose, serine, alanine, histidine, proline, fatty acids | [10-12] |
| *Staphylococci* | 50 | Glucose, serine, threonine, glycine, alanine, histidine, proline, valine, leucine, isoleucine | [13-15] |
| *B. subtilis* | 24 | Glucose, alanine, histidine, proline, malate | [6, 16-19] |

Amounts of glucose, amino acids, and fatty acids initially on the skin and their replenishing rates (**Table S3**) were calculated from reports of values extracted from the forearm of human volunteers [3, 4]. For simplicity, fatty acids were assumed to be saturated with a chain length of 16 carbons [20]. Carbon sources in LB media (**Table S4**) were determined based on reported values detected by HPLC [21]. Energy numbers in the model refer to the increase in energy gained from eating a given nutrient and were determined based on the net yield of ATP generated from complete catabolism through the TCA cycle, assuming ratios of 2.5 NADH per ATP and 1.5 FADH_2_ per ATP [22]. Using values obtained from literature to provide initial estimates, carbon source starting amounts and replenishing rates were then adjusted by increasing their values 10-fold and energy numbers were reduced 10-fold to prevent complete death or overgrowth of populations in the model (**Table S3**), following an approach similar to that taken in the Gutlogo model [2]. For each simulation, the model was run three times, and the average and standard deviation across three simulations was reported.

**Table S3.** Carbon sources in model

| **Carbon source** | **Starting amount** (nmol/cm^2^) | **Replenishing rate** (nmol/cm^2^/min) | **Energy** (ATP) | **References** |
| --- | --- | --- | --- | --- |
| Glucose | 12 | 0.01 | 3.2 | [3, 22] |
| Serine | 3700 | 4.46 | 1.25 | [3, 23] |
| Threonine | 840 | 1.00 | 1.9 | [3, 23] |
| Glycine | 2500 | 3.42 | 1.25 | [3, 23] |
| Alanine | 1840 | 2.08 | 1.25 | [3, 23] |
| Histidine | 1010 | 1.04 | 2.25 | [3, 23] |
| Proline | 420 | 0.46 | 2.75 | [3, 23] |
| Leucine/Isoleucine | 330 | 0.31 | 3.3 | [3, 23] |
| Valine | 300 | 0.33 | 2.75 | [3, 23] |
| Fatty acids | 1170 | 4.46 | 10.6 | [4, 22] |
| Phenylalanine/ Tyrosine | 295 | 0.31 | 3.0 | [3, 23] |
| Malate | n/a | n/a | 1.0 | [22] |

**Table S4.** Carbon sources in LB media

| **Carbon source** | **Amount** (nmol/cm^2^) | **Reference** |
| --- | --- | --- |
| Glucose | 0 | [21] |
| Serine | 1821 | [21] |
| Threonine | 1571 | [21] |
| Glycine | 1429 | [21] |
| Alanine | 2071 | [21] |
| Histidine | 500 | [21] |
| Proline | 3393 | [21] |
| Leucine/Isoleucine | 5071 | [21] |
| Valine | 2500 | [21] |
| Fatty acids | 0 | [21] |
| Phenylalanine/ Tyrosine | 1786 | [21] |

**Table S5.** DNA primers

| Name | Sequence (5’-3’)^1^ |
| --- | --- |
| RBS0_GFP_5p.F | aatttgcaagcttaaggaggacaaacatgagcaaaggtgaagaactg |
| RBS1_GFP_5p.F | cttagaggtggtgtacaaacatgagcaaaggtgaagaactg |
| RBS2_GFP_5p.F | cttaagaggagataacaaacatgagcaaaggtgaagaactg |
| RBS3_GFP_5p.F | cttaaggagagataacaaacatgagcaaaggtgaagaactg |
| RBS4_GFP_5p.F | atattaagaggaggagacaaacatgagcaaaggtgaagaactg |
| RBS5_GFP_5p.F | cttaaaggaggtgtacaaacatgagcaaaggtgaagaactg |
| GFP_3p.R | gagtcgacctgcaggcatgcttatttttcgaactgcggatgg |
| RB_5p.F | atccgcagttcgaaaaataagcatgcctgcaggtcg |
| RBS0_RB_3p.R | agttcttcacctttgctcatgtttgtcctccttaagcttgcaaattatatcaacg |
| RBS1_RB_3p.R | catgtttgtacaccacctctaagcttgcaaattatatcaacgttaataagac |
| RBS2_RB_3p.R | catgtttgttatctcctcttaagcttgcaaattatatcaacgttaataagac |
| RBS3_RB_3p.R | catgtttgttatctctccttaagcttgcaaattatatcaacgttaataagac |
| RBS4_RB_3p.R | ttgtctcctcctcttaatataagcttgcaaattatatcaacgttaataagac |
| RBS5_RB_3p.R | catgtttgtacacctcctttaagcttgcaaattatatcaacgttaataagac |

^1^Ribosome binding sites are underlined

**LD_50_ cytotoxicity assay**

Wells were read using a Biotek Synergy H4 Hybrid Microplate Reader according to the assay protocol, either with absorbance at 570 nm and 600 nm or with fluorescence using a 560 nm excitation wavelength and 590 nm emission wavelength. For absorbance measurements, the percent reduction of alamarBlue reagent was calculated according to the assay protocol using the equation 100*(ε_2_A_1_ – ε_1_A_2_)/( ε_2_A’_1_ – ε_1_A’_2_), where ε_1_ and ε_2_ are the molar extinction coefficients of alamarBlue at 570 nm and 600 nm, respectively, A_1_ and A_2_ are the absorbances of the treated wells at 570 nm and 600 nm, respectively, and A’_1_ and A’_2_ are the absorbances of the negative toxicity control at 570 nm and 600 nm, respectively. The average value for the positive toxicity control (2% Tween 20, expected to correspond to complete cell death) was subtracted from all measurements, and the resulting values were normalized by the negative toxicity (no treatment) control. The calculated values were plotted and fitted to a line using a simple linear regression, and LD_50_ was calculated as the concentration that corresponded to 50% cell survival.


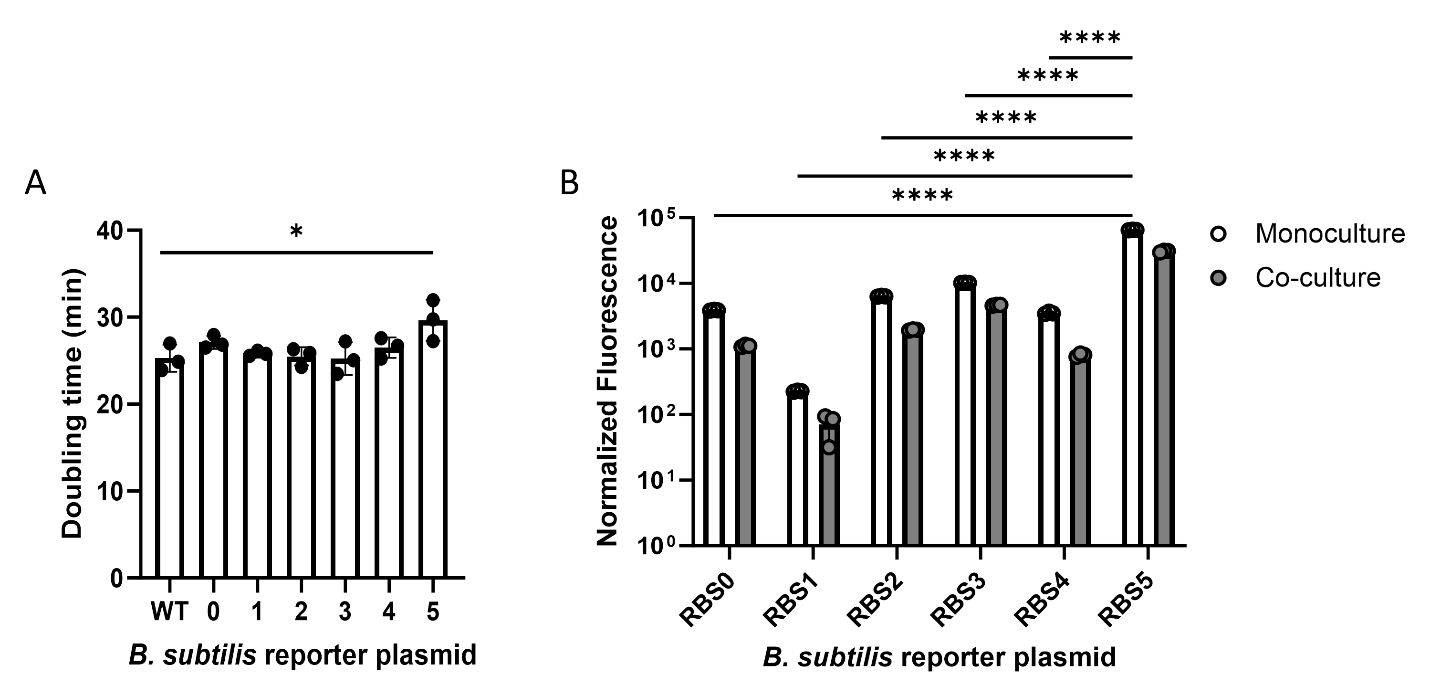


**Figure S1.** Survival and production of heterologous protein by *B. subtilis* in culture. (A) Doubling times for wild-type (WT) *B. subtilis* and strains harboring plasmids pGFP-RBS0 through pGFP-RBS5. * p < 0.05, one-way ANOVA comparing doubling times to wild-type. (B) Fluorescence of GFP expressed in B*. subtilis* strains harboring different reporter plasmids in monoculture and in co-culture with parent strain *B. subtilis* 168. Data show mean +/- standard deviation of three replicates. **** p < 0.0001, one-way ANOVA comparing fluorescence values in monoculture.


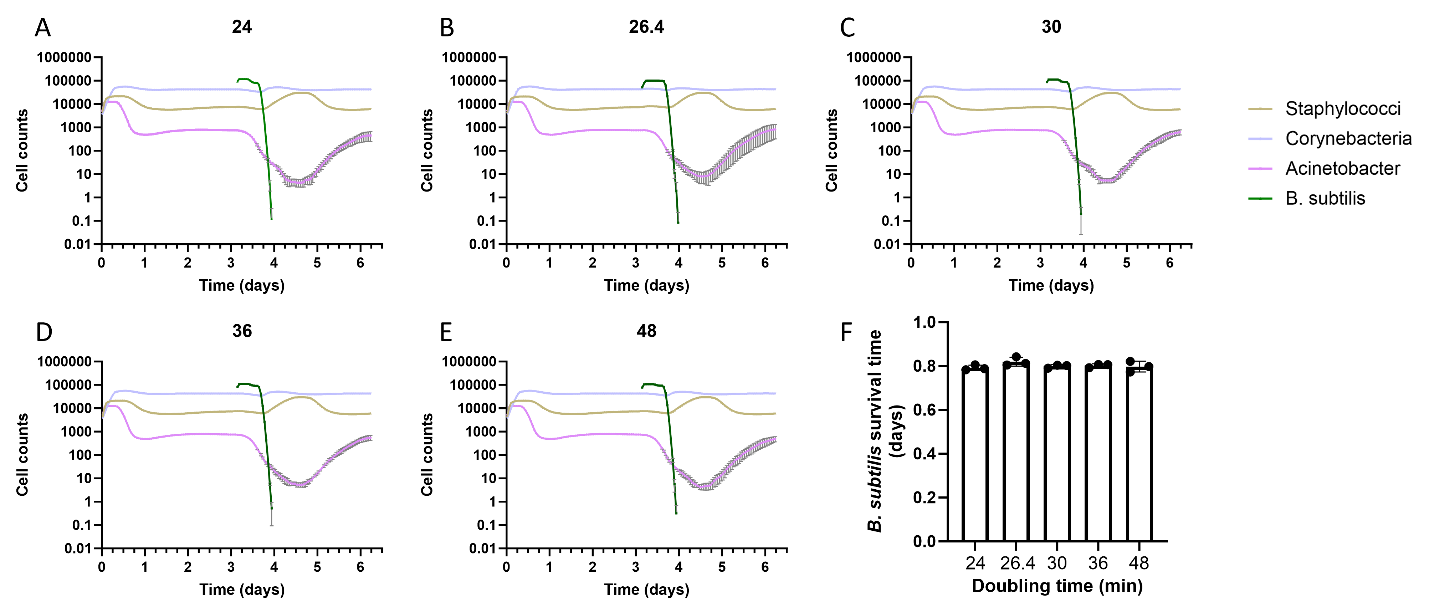


**Figure S2**. Simulation of skin bacteria population dynamics with different doubling times for added *B. subtilis* species. (A) 24 min; (B) 26.4 min; (C) 30 min; (D) 36 min; and (E) 48 min, representing a 0%, 10%, 25%, 50%, and 100% increase to doubling time, respectively. (F) Survival time of *B. subtilis* with different doubling times. Lines and bars show mean +/- standard deviation of 3 replicate simulations.


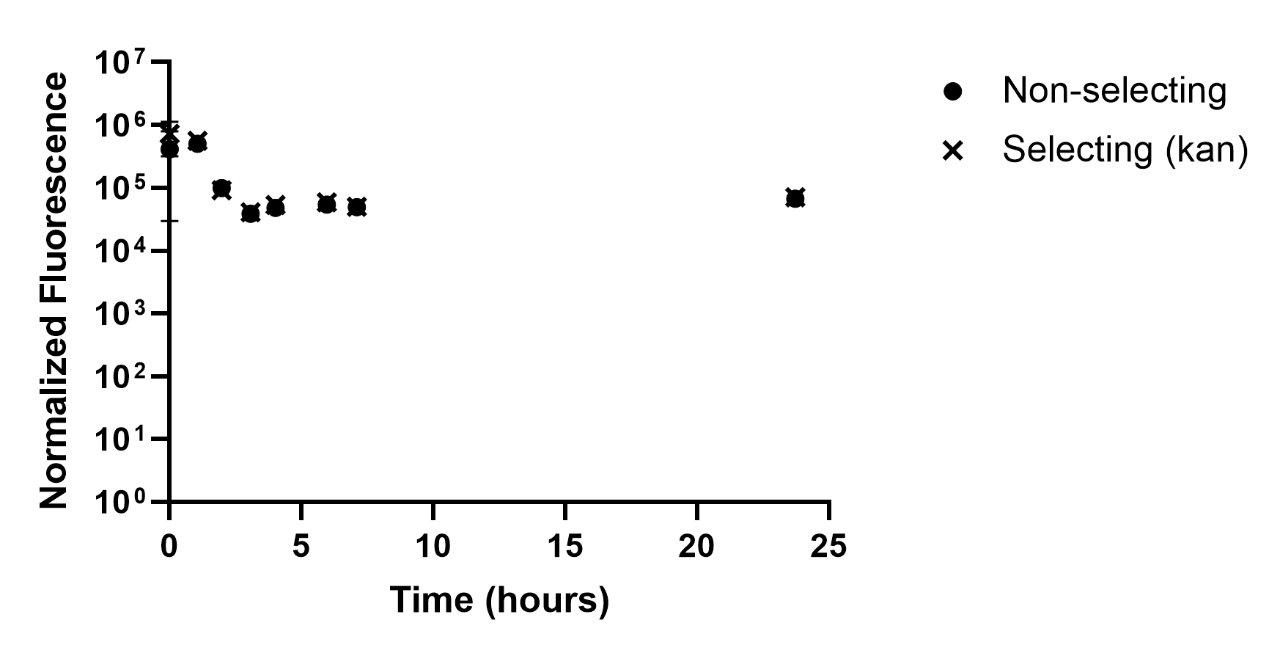


**Figure S3.** Stability of plasmid pGFP-RBS5 in *B. subtilis*. Plasmid-harboring cells were grown in LB liquid media either with 10 µg/ml kanamycin (selecting) or without antibiotic (non-selecting). Fluorescence was measured using a fluorescence plate reader over 24 hours.


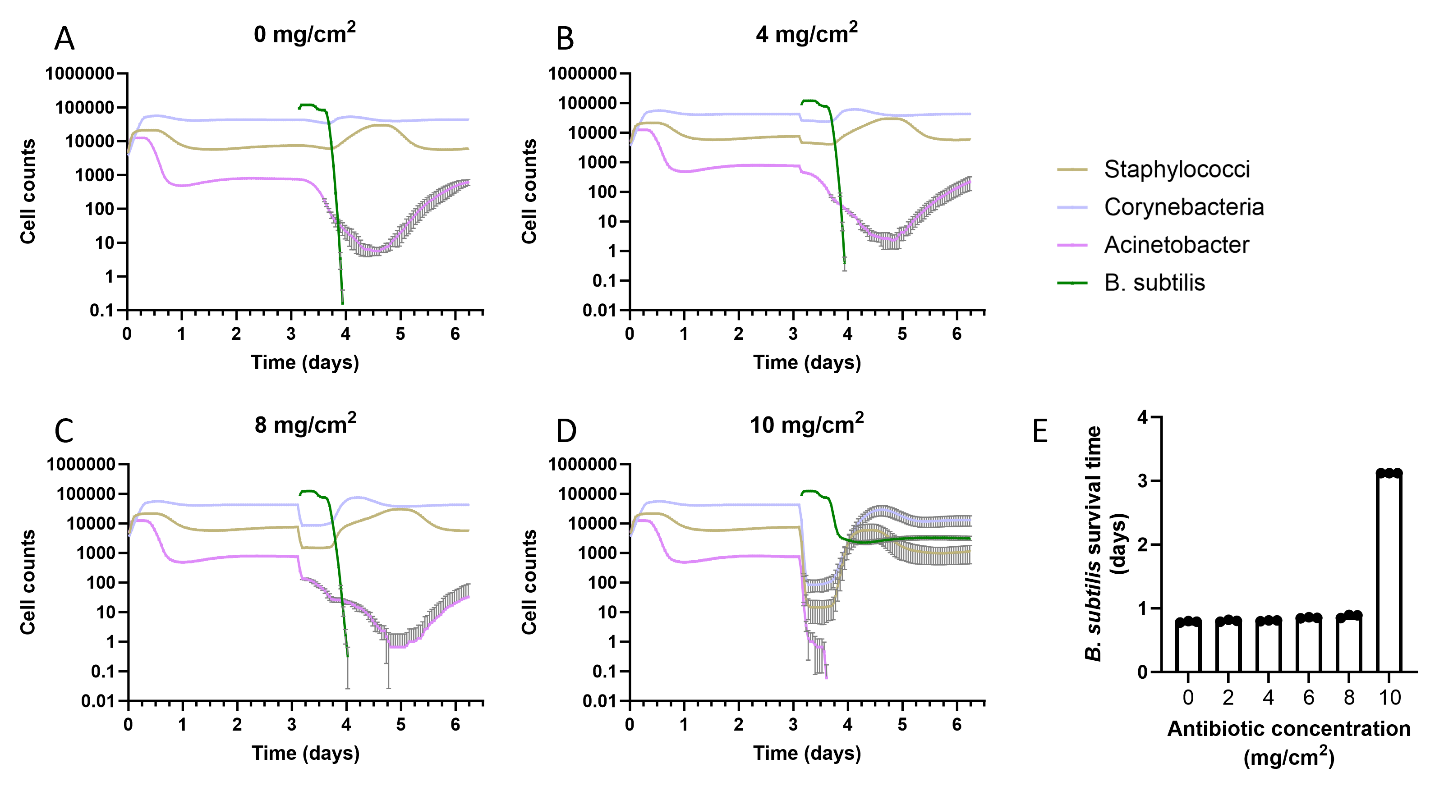


**Figure S4.** Simulation of skin bacteria population dynamics when adding antibiotics concurrently with *B. subtilis* in LB media. (A) 0 mg/cm^2^; (B) 4 mg/cm^2^; (C) 8 mg/cm^2^; (D) 10 mg/cm^2^ of antibiotic. (E) Survival time of *B. subtilis* with different concentrations of antibiotic. At 10 mg/cm^2^, *B. subtilis* appeared to survive long-term, but is shown as 3 days, because the simulation ended at that point. Lines and bars show mean +/- standard deviation of 3 replicate simulations.


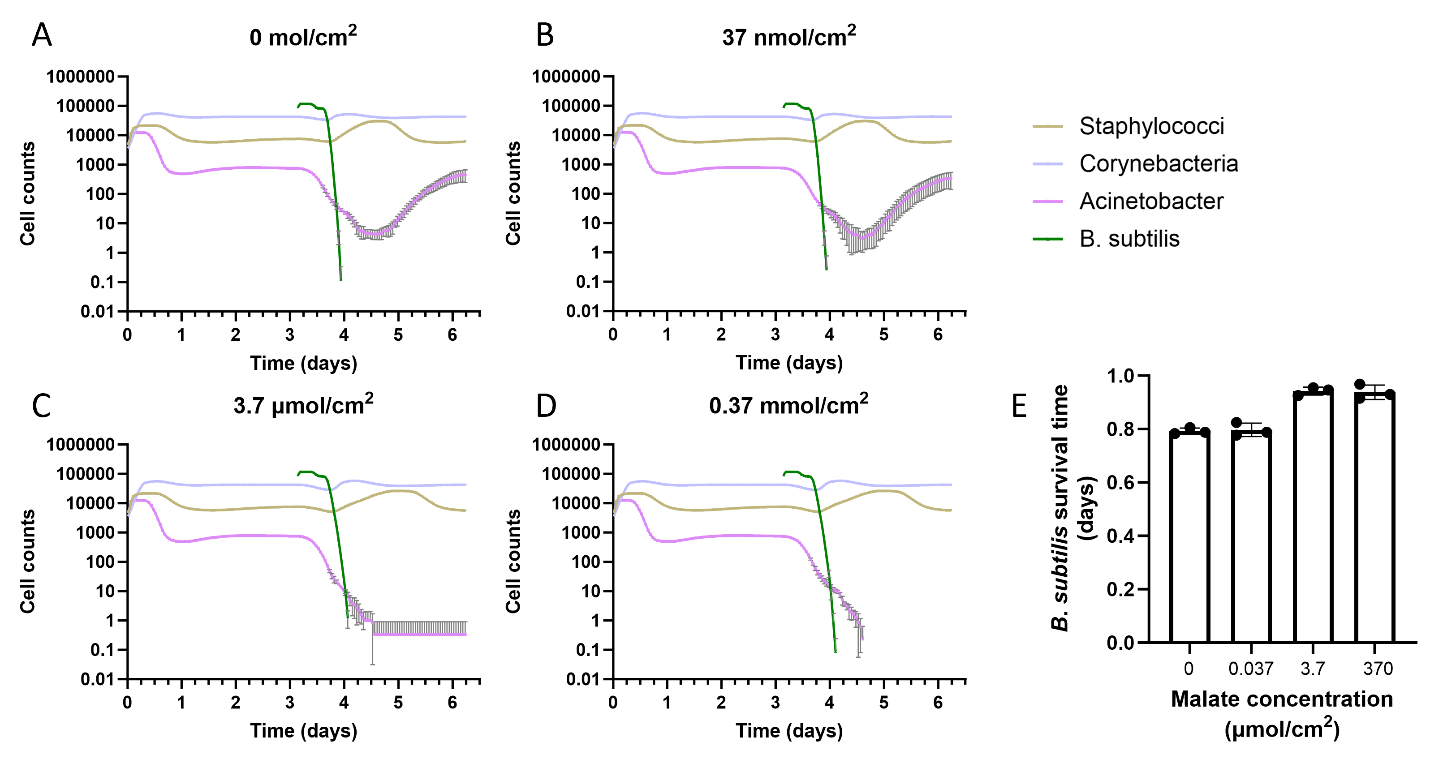


**Figure S5.** Simulation of skin bacteria population dynamics when adding supplemental carbon source concurrently with *B. subtilis* in LB media. (A) 0 mol/cm^2^ malate added; (B) 37 nmol/cm^2^ malate; (C) 3.7 umol/cm^2^ malate; (D) 0.37 mmol/cm^2^ malate. Lines represent mean values and error bars show standard deviation across 3 simulations. In most cases, the error bars are smaller than the line thickness. (E) survival time of *B. subtilis* with different concentrations of carbon source. Data show mean +/- standard deviation of 3 replicate simulations.

**
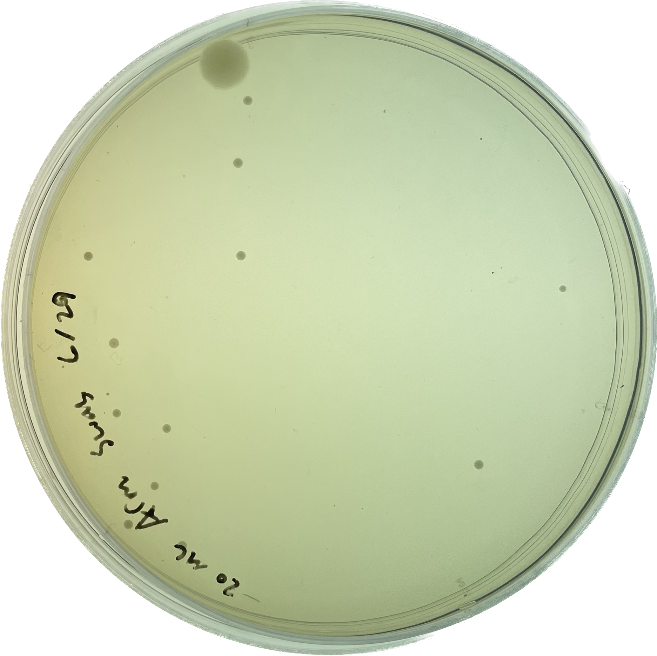
**

**Figure S6.** Bacterial load of human skin swab sample plated on non-selecting LB agar. This study used the same volume (20 µL) of human skin swab sample that was applied to the human skin tissue culture (Figure 7).

**Figure S7.** Cytotoxicity of bacterial supernatants against HaCaT human keratinocyte cells *in vitro*. Dotted lines show 95% confidence intervals for linear regression analysis. Data points show mean +/- standard deviation of 8 replicates.

**References**

1. Gary An QM, Joyeeta Dutta-Moscato, Yoram Vodovotz. Agent-based models in translational systems biology. WIREs Systems Biology and Medicine. 2009;1.

2. Lin C, Culver J, Weston B, Underhill E, Gorky J, Dhurjati P. GutLogo: Agent-based modeling framework to investigate spatial and temporal dynamics in the gut microbiome. PLoS One. 2018;13(11):e0207072.

3. Sylvestre JP, Bouissou CC, Guy RH, Delgado-Charro MB. Extraction and quantification of amino acids in human stratum corneum in vivo. Br J Dermatol. 2010;163(3):458-65.

4. Greene RS, Downing DT, Pochi PE, Strauss JS. Anatomical variation in the amount and composition of human skin surface lipid. J Invest Dermatol. 1970;54(3):240-7.

5. Zhan Gao C-hT, Zhiheng Pei, Martin J Blaser. Molecular analysis of human forearm superficial skin bacterial biota. PNAS. 2007;104.

6. Meyer FM, Stulke J. Malate metabolism in Bacillus subtilis: distinct roles for three classes of malate-oxidizing enzymes. FEMS Microbiol Lett. 2013;339(1):17-22.

7. Rudrappa T, Czymmek KJ, Pare PW, Bais HP. Root-secreted malic acid recruits beneficial soil bacteria. Plant Physiol. 2008;148(3):1547-56.

8. Antunes LC, Imperi F, Carattoli A, Visca P. Deciphering the multifactorial nature of Acinetobacter baumannii pathogenicity. PLoS One. 2011;6(8):e22674.

9. Philippe J M Bouvet PADG. Taxonomy of the Genus Acinetobacter with the Recognition of Acinetobacter baumannii sp. nov., Acinetobacter haemolyticus sp. nov., Acinetobacter johnsonii sp. nov., and Acinetobacter junii sp. nov. and Emended Descriptions of Acinetobacter calcoaceticus and Acinetobacter lwoffii International Journal of Systemic Bacteriology. 1986;36.

10. Brune I, Becker A, Paarmann D, Albersmeier A, Kalinowski J, Puhler A, et al. Under the influence of the active deodorant ingredient 4-hydroxy-3-methoxybenzyl alcohol, the skin bacterium Corynebacterium jeikeium moderately responds with differential gene expression. J Biotechnol. 2006;127(1):21-33.

11. Mounier J, Rea MC, O'Connor PM, Fitzgerald GF, Cogan TM. Growth characteristics of Brevibacterium, Corynebacterium, Microbacterium, and Staphylococcus spp. isolated from surface-ripened cheese. Appl Environ Microbiol. 2007;73(23):7732-9.

12. Tauch A, Kaiser O, Hain T, Goesmann A, Weisshaar B, Albersmeier A, et al. Complete genome sequence and analysis of the multiresistant nosocomial pathogen Corynebacterium jeikeium K411, a lipid-requiring bacterium of the human skin flora. J Bacteriol. 2005;187(13):4671-82.

13. Oliveira F, Franca A, Cerca N. Staphylococcus epidermidis is largely dependent on iron availability to form biofilms. Int J Med Microbiol. 2017;307(8):552-63.

14. Halsey CR, Lei S, Wax JK, Lehman MK, Nuxoll AS, Steinke L, et al. Amino Acid Catabolism in Staphylococcus aureus and the Function of Carbon Catabolite Repression. mBio. 2017;8(1).

15. Liu YK, Kuo HC, Lai CH, Chou CC. Single amino acid utilization for bacterial categorization. Sci Rep. 2020;10(1):12686.

16. Bottinger B, Semmler F, Zerulla K, Ludt K, Soppa J. Regulated ploidy of Bacillus subtilis and three new isolates of Bacillus and Paenibacillus. FEMS Microbiol Lett. 2018;365(4).

17. Kin-Ichi Sugae EF. Requirement for Acetate and Glycine (or Serine) for Sporulation without Growth of Bacillus subtilis. Journal of Bacteriology. 1970;104(3).

18. Boris R Belitsky ALS. Role and Regulation of Baillus subtilis Glutamate Dehydrogenase Genes Journal of Bacteriology. 1998;180.

19. Chasin LA, Magasanik B. Induction and Repression of the Histidine-degrading Enzymes of Bacillus subtilis. Journal of Biological Chemistry. 1968;243(19):5165-78.

20. M N A Ansari NN, H C Fu. Fatty acid composition of the living layer and stratum corneum lipids of human sole skin epidermis Lipids 1970;5.

21. Sezonov G, Joseleau-Petit D, D'Ari R. Escherichia coli physiology in Luria-Bertani broth. J Bacteriol. 2007;189(23):8746-9.

22. Albert Lehninger DN, Michael Cox Lehninger Principles of Biochemistry. . 4 ed: W. H. Freeman 2005.

23. Bender DA. The metabolism of "surplus" amino acids. Br J Nutr. 2012;108 Suppl 2:S113-21.
